# The mTOR inhibitor rapamycin down-regulates the expression of the ubiquitin ligase subunit Skp2 in breast cancer cells

**DOI:** 10.1186/bcr1533

**Published:** 2006-07-19

**Authors:** Ma'anit Shapira, Eli Kakiashvili, Tzur Rosenberg, Dan D Hershko

**Affiliations:** 1 Department of Surgery A, Rambam Medical Center, Haifa 31096, Israel; 2 Technion – Israel Institute of Technology, Haifa 31096, Israel; 3 Department of Surgery B, Rambam Medical Center, Haifa 31096, Israel; 4 Breast Health Institute, Rambam Medical Center, Haifa 31096, Israel

## Abstract

**Introduction:**

Loss of the cyclin-dependent kinase inhibitor p27 is associated with poor prognosis in breast cancer. The decrease in p27 levels is mainly the result of enhanced proteasome-dependent degradation mediated by its specific ubiquitin ligase subunit S phase kinase protein 2 (Skp2). The mammalian target of rapamycin (mTOR) is a downstream mediator in the phosphoinositol 3' kinase (PI3K)/Akt pathway that down-regulates p27 levels in breast cancer. Rapamycin was found to stabilize p27 levels in breast cancer, but whether this effect is mediated through changes in Skp2 expression is unknown.

**Methods:**

The expression of Skp2 mRNA and protein levels were examined in rapamycin-treated breast cancer cell lines. The effect of rapamycin on the degradation rate of Skp2 expression was examined in cycloheximide-treated cells and in relationship to the anaphase promoting complex/Cdh1 (APC\C) inhibitor Emi1.

**Results:**

Rapamycin significantly decreased Skp2 mRNA and protein levels in a dose and time-dependent fashion, depending on the sensitivity of the cell line to rapamycin. The decrease in Skp2 levels in the different cell lines was followed by cell growth arrest at G1. In addition, rapamycin enhanced the degradation rate of Skp2 and down-regulated the expression of the APC\C inhibitor Emi1.

**Conclusion:**

These results suggest that Skp2, an important oncogene in the development and progression of breast cancer, may be a novel target for rapamycin treatment.

## Introduction

Loss of p27, an inhibitor of cyclin-dependent kinases (Cdks), commonly occurs in malignant diseases and may have a profound impact on the rate of tumor progression and patients' clinical outcome (reviewed in Tsihlias and colleagues [1]). Studies have shown that the decrease in p27 levels in these cancers is mainly the result of its rapid degradation by the ubiquitin-proteasome pathway rather than from decreased protein synthesis or gene mutation [2,3]. The machinery involved in targeting p27 for degradation is an SCF (Skp1-Cullin-F-box protein)-type ubiquitin ligase complex that contains S phase kinase protein (Skp)2 as the specific substrate recognizing subunit [4,5]. SCF complexes belong to a large family of ubiquitin ligases that contain several constant subunits (called Cullin-1, Skp1 and ROC1) and a variable subunit known as an F-box protein [6]. Each F-box protein binds a specific subset of protein substrates and thus promotes their ligation to ubiquitin and subsequent degradation by the proteasome [7].

Skp2 is an F-box protein that was originally discovered, along with Skp1, as a protein associated with the S-phase kinase Cdk2/cyclin A and hence its name. The role of Skp2 as the main rate limiting regulator for the degradation of p27 has been clearly shown in several human cancers, including breast cancer [8-11]. Moreover, tumors overexpressing Skp2 were strongly associated with low p27 levels and poor disease-free and overall survival [9-11]. The exact mechanisms that promote Skp2 overexpression in these cancers are at present not well understood. It was suggested that Skp2 acts as an oncogene in breast cancer and thus is overexpressed by increased transcriptional activity [11]. However, more recent *in vitro *studies have discovered that Skp2 is also regulated by its rate of protein degradation, which by itself is mediated by the ubiquitin-proteolytic system [12,13]. These studies have found that the specific ubiquitin ligase that targets Skp2 for degradation is the anaphase promoting complex/Cdh1 (APC/C). However, the role of APC/C activity in the regulation of Skp2 levels in human cancers is at present unknown.

Some studies have shown that alternative cellular mechanisms may also be involved in p27 deregulation in cancer. For example, constitutive activation of phosphoinositol 3' kinase (PI3K) and its effector protein kinase B (PKB/Akt) down-regulate p27 nuclear levels by either repressing its transcription through Akt phosphorylation of forkhead transcription factors or by impairing nuclear import, leading to cytoplasmic accumulation of p27 [14,15]. Activation of this pathway commonly occurs in breast cancer and may arise through oncogenic receptor tyrosine kinase activation, mutational loss of PTEN (phosphatase and tensin homologue deleted from chromosome 10), or through activating mutation of PKB/Akt [16,17]. It can also be activated by membrane receptors, including the HER family of growth factor receptors, the insulin-like growth factor receptors or by estrogen receptors [18-20]. Some studies have suggested that PI3K/Akt activation may also affect the rate of p27 proteolysis in some human cancers [21-23]. In multiple myeloma, for example, inhibition of the PI3K/Akt pathway by LY294002 resulted in p27 accumulation, which, in turn, was associated with a decrease in Skp2 levels [23]. However, the mechanisms that down-regulate Skp2 expression by inhibition of this pathway in multiple myeloma or in any other cancer are at present unknown.

The mammalian target of rapamycin (mTOR; also known as FRAP, RAFT1 and RAPT1) is a downstream effector of the PI3/Akt pathway that has recently received great attention as a potential novel therapeutic modality for the treatment of breast cancer. Rapamycin and its synthetic analogues target mTOR by binding to immunophilin FK506 binding protein 12, thereby inhibiting signals required for cell cycle progression and cell growth [24,25]. By inhibiting mTOR, it inactivates both the 40S ribosomal protein (S6K1) and 4E-binding protein 1 (4E-BP1), which are important for translation of specific mRNA involved in cell cycle progression, and thus lead to growth arrest at G1. In clinical trials, treatment with either rapamycin or its analogue CCI-779 have shown remarkable anticancer activities in some patients, but others did not respond [26,27]. Recent studies explored the determinants of sensitivity of breast cancer cell lines to rapamycin, and discovered that cells that express high levels of activated Akt or S6K1 were also highly sensitive to rapamycin [28]. It was also found that in rapamycin-sensitive cells p27 levels were up-regulated, but whether this was caused by altering Skp2-dependent degradation was not examined.

In the present study, we examined the effects of rapamycin on Skp2 expression in breast cancer lines and the regulatory mechanisms that determine its cellular abundance. Our results suggest that rapamycin down-regulates Skp2 expression in cultured breast cancer cell lines by interfering with gene transcription as well as by increasing its rate of protein degradation.

## Materials and methods

### Cell cultures and transfections

Human breast cancer cell lines T47D and MDA-MB-231 were provided by Dr H Degani (Weizmann Institute of Science, Rehovot, Israel). Because Skp2 levels change during the cell cycle (being normally highest in the S-phase and lowest in G1 phase) we cultured the cells in different media under conditions of similar proliferation rates in both cell lines. MDA-MB-231 cells were grown in RPMI medium (Biological Industries, Beth Ha'emek, Israel) supplemented with 10% fetal calf serum, 100 Units penicillin and 100 μg streptomycin per ml and 1 mM sodium pyruvate. T47D cells were cultured in a similar medium that also contained 10 μg/ml insulin. Both cell lines were cultured at 37°C in 5% CO_2_. Under these conditions, the proliferation rate of all cell lines was similar (21 to 22 h doubling time). For transfection studies, T47D cells were seeded at a density of 5 × 10^5 ^cells per 60 mm petri dish and transfected with either pcDNAIII empty vector or Flag-tagged Skp2 in pcDNAIII vector (2 μg) using FuGENE 6 reagent (Roche Applied Science, Mannheim, Germany).

### Proliferation assays

Cells were seeded in 24-well plates at a concentration of 1 × 10^4 ^cells/per well for 24 h and then treated with different concentrations of rapamycin or DMSO. Cells were then detached from the wells at different time points (12 to 72 h) by trypsin and counted by hemocytometry.

### Protein extract preparation

Cells were grown in 10 cm dishes until 80% confluence was reached before use. They were harvested into ice-cold PBS and pelleted by centrifugation (200 g for 3 minutes). Cells were then suspended in one packed-cell volume of lysis buffer containing 50 mM Tris-HCl pH 7.6, 250 mM NaCl, 10 mM EDTA, 0.5% Nonidet P-40, 50 mM NaF, 10 μg/ml leupeptin, 10 μg/ml chymostatin, 10 μg/ml pepstatin, 2 mM N-ethylmaleimide, 1 mM Phenylmethanesulfonyl fluoride (PMSF) and 1:100 protease inhibitor cocktail (Sigma Aldrich, Rehovot, Israel), incubated on ice for 30 minutes and centrifuged again at 20,000 g for 15 minutes. Protein concentrations were determined by the Bradford assay (Bio-Rad Laboratories, Hercules, CA, USA) using bovine albumin as the standard.

### Western blot analysis

Aliquots containing 30 μg protein were resolved by electrophoresis on a 12% SDS-polyacrylamide gel and transferred to nitrocellulose membranes. The membranes were probed with mouse monoclonal antibody directed against either Skp2 (Zymed Inc., San Francisco, CA, USA) at 1:500, p27 (Transduction Laboratories, Lexington, KY, USA) at 1:1000 or the polyclonal rabbit early mitotic inhibitor 1 (Emi1; gift of Dr Pagano, New York University, NY, USA) at 1:250. The same nitrocellulose membranes were also probed with a mouse monoclonal antibody directed against Skp1 (1:1000 dilution; Transduction Laboratories). Because levels of Skp1 do not change in the cell cycle, this protein served as an internal control for normalization with respect to the loading of cellular protein. To detect phosphorylated proteins in the mTOR pathway we used rabbit polyclonal antibodies against phospho-4E-BP1 (Thr37/46) or phospho-p70 S6 kinase (Thr389) (Cell Signaling Technology Inc., Beverly, MA, USA) diluted at 1:1000. For the latter antibodies, bovine serum albumin instead of dry milk was used in blocking buffer and antibody solutions. After washing with Tris Buffer Saline with 0.1% Tween 20 (TBST), the immunoreactive proteins were visualized with HRP-conjugated secondary antibody (Pierce Biotechnology, Rockford, IL, USA) at 1:10,000, and by enhanced chemiluminescence (SuperSignal West Pico; Pierce Biotechnology). All blots were repeated at least twice. Protein levels were quantified with ImageMaster VSD-CL (Rhenium, Jerusalem, Israel) using Bio Imaging System 303PC software (DNR Bio Imaging Systems, Jerusalem, Israel). Analyses were done using TINA 2.1 software (Raytest, Straubenhardt, Germany).

### RNA extraction and real-time RT-PCR

Total RNA was extracted by a modification of the acid guanidinium thiocyanate-phenol-chloroform method using Tri-Reagent solution (Molecular Research Center, Cincinnati, OH, USA) according to the manufacturer's instructions. Final pellets were dissolved in 40 μl RNase-free water with 1 u/μl RNasin (Promega, Medison, WI). RNA quantification was performed using spectrophotometry and samples were diluted to 0.5 μg/μl concentration. The quality of RNA was determined by loading 2 μg on RNA-agarose gel (1.2%) and fine concentration corrections were made using UVIgelstarMw software (UVItec, Cambridge, UK). Only intact RNA was used for further experiments.

Quantitative real-time reverse-transcription PCR (RT-PCR) analyses for mRNA were performed using Rotor Gene 2000 real time cycler instrument and software (Corbett, Australia) with a QuantiTec SYBR Green RT-PCR kit (Qiagen, Valencia, CA, USA). Phosphoglycerate kinase (PGK), a housekeeping gene, was chosen as an internal standard to control for variability in amplification. For each condition, duplicate test tubes containing 100 ng of total RNA and 400 nM Skp2 or PGK gene primers in a total volume of 25 μl were used. The primers used were: Skp2, sense primer GCTGCTAAAGGTCTCTGGTGT and antisense primer AGGCTTAGATTC TGCAACTTG; PGK, sense primer TTTAAGGGTTCCTGGCACTG, antisense primer CAGTTTGGAGCTCCTGGAAG. These resulted in one product of either 292 or 200 base-pairs (bp) with Tm of 81°C and 83°C for Skp2 and PGK genes, respectively. Reaction profiles used were 35 cycles of 95°C for 5 s, 60°C for 20 s and 72°C for 15 s, followed by melting of 72 to 90°C. The number of copies was drawn from a standard curve of 10^3 ^to 10^7 ^copies/μl for each gene separately, and levels of expression were calculated as the ratio between Skp2 and PGK copies in each RNA sample.

### Fluorescence-activated cell sorting

Cells were treated with rapamycin (20 nM) or DMSO (0.02%) for 24 h, then trypsinized, resuspended in media and spun down for 5 minutes at 200 g. Cells were then washed with PBS, and fixed at a final concentration of 10^6 ^to 10^7 ^cells/ml in 70% ethanol. Samples were kept at 4°C until staining. Fixed cells were incubated with 100 μl of RNAse 1 mg/ml for 30 minutes at 37°C, followed by 30 minutes incubation with 1 ml of 50 μg/ml propidium iodide in PBS. Cells were counted on a FACSCalibur cell sorter using CellQuest software (Beckton Dickinson, Mountain View, CA, USA). Cell cycle analysis was preformed by a commercial DNA analysis package (Modfit LT 2.0, Verity Software House Inc., Topsham, ME, USA), and the percentages of cells in the G1, S, and G2/M phases of the cell cycle were determined.

### Degradation assays

To assess the degradation rate of Skp2 in rapamycin-treated and untreated cells, cells were seeded at a concentration of 1.2 × 10^6 ^cells per dish, cultured for 24 h and then treated with rapamycin (20 nM) or DMSO (0.02%) for another 24 h. Cycloheximide (100 μg/ml) was then added to the medium. Cells were collected at different time points (1 to 4 h) and protein extracts were prepared as described above. Skp2 levels and half-life decay were quantified by immunoblot analyses as described above.

## Results

**Figure 1:**
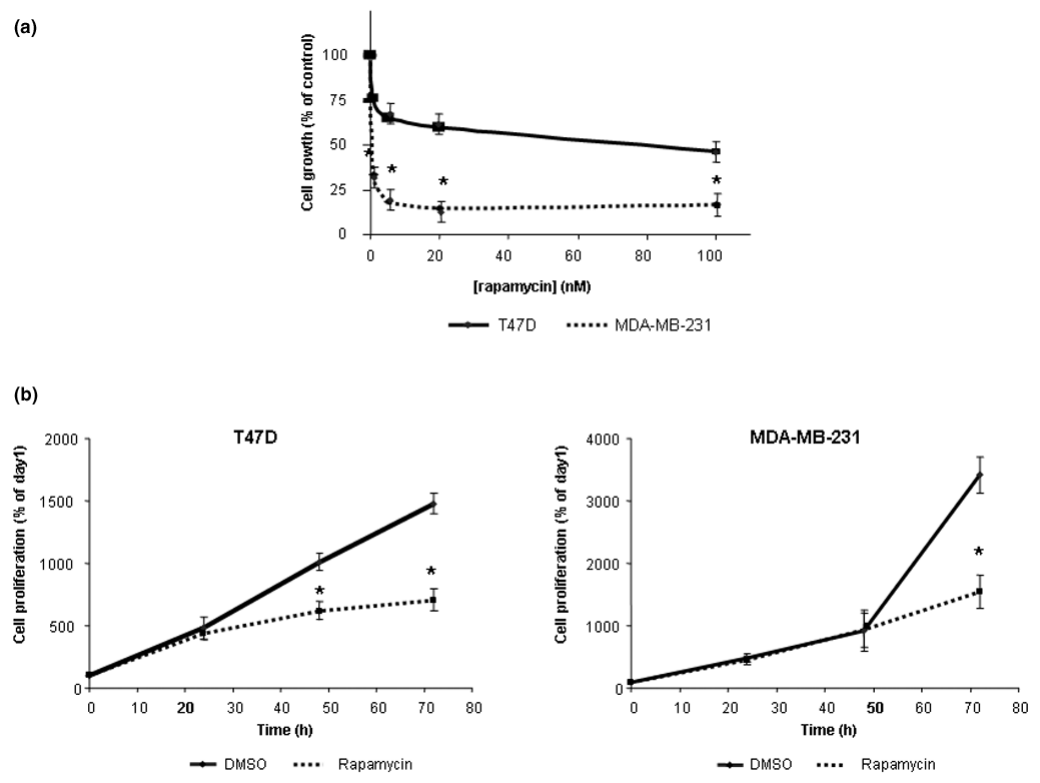
Effect of rapamycin on cell growth. (**a**) The dose-effect curve of rapamycin on cell growth. The breast cancer cell lines T47D and MDA-MB-231 were treated with different concentrations of rapamycin for 72 h and the decrease in growth rate compared to control cells was assessed. (**b**) Time dependence of the effect of rapamycin on cell growth. The breast cancer cell lines T47D (left) and MDA-MB-231 (right) were treated with rapamycin (20 nM) for different time periods and the decrease in growth rate compared to control cells was assessed. Results are means ± standard error of the mean, with *n *= 3 for each treatment. *p < 0.05 between the two conditions.

**Figure 2:**
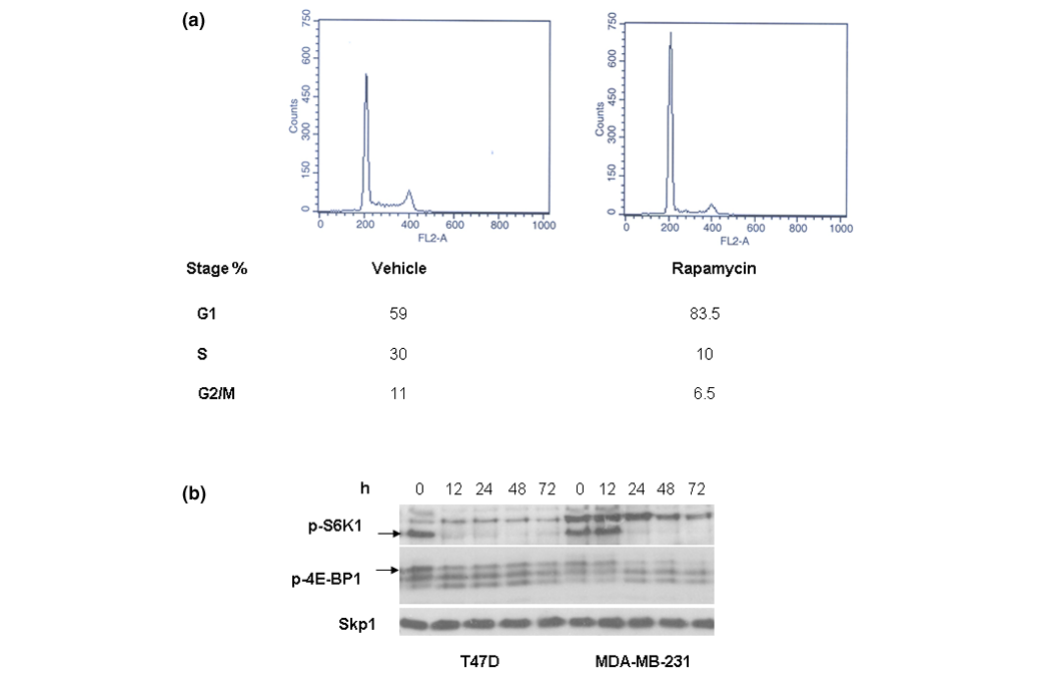
The effect of rapamycin on cell cycle profile and mTOR activation. (**a**) The effect of rapamycin on cell cycle distribution. T47D cells were treated with rapamycin (20 nM) or DMSO (0.02%) for 24 h and subjected to FACS analysis to determine the distribution of the cell cycle. (**b**) The effect of rapamycin on the phosphorylation of the mTOR effectors p-S6K1 and p-4E-BP1. The breast cancer cell lines T47D and MDA-MB-231 were treated with rapamycin (20 nM) for different time periods and the phosphorylated forms of the proteins was determined by western blot analysis. Skp1 was used as a loading control. Arrows indicate the specific bands.

To examine the dose-effect of rapamycin treatment on cellular growth rate in different breast carcinoma cell lines, cells were exposed to different concentrations of rapamycin for 72 h. A significant decrease in cell growth rate was observed after exposure to 5 nM of rapamycin in both cell lines and this effect was maximal at 20 nM in MDA-MB-231 cells and at 100 nM in T47D cells (Figure 1a). To examine the time-dependence effect of rapamycin on cell proliferation, cells were treated with rapamycin (20 nM) for different time periods and the effect on growth rate was assessed. Following an initial delay, a significant inhibitory effect on cell growth became evident at 24 h for T47D cells and after 48 h for the MDA-MB-231 cells, and this effect was further increased up to 72 h (Figure 1b). The cell cycle inhibitory effect of rapamycin (20 nM), as determined by fluorescence-activated cell sorting (FACS) analysis, resulted in a significant proportion of cells arrested at G1 (83.5% compared to 59% in vehicle treated cells) (Figure 2a). To determine the inhibitory effect of rapamycin on mTOR function in these experimental conditions, we examined the inactivation of its two major downstream signaling components p70S6 kinase (S6K1) and 4E-BP1. Cells were treated with rapamycin at a concentration of 20 nM for 24 h and subjected to western blot analysis to determine phospho-S6K1 and phospho-4E-BP1 protein levels. Levels of the phosphorylated forms of both proteins were markedly decreased by rapamycin at 12 h in T47D cells and at 24 h in MDA-MB-231 cells, but this effect was stronger in both cell lines for S6K1 (Figure 2b). Thus, the inhibitory effect on cell growth was associated with direct inhibition of the phosphorylation of mTOR target proteins S6K1 and 4E-BP1.

**Figure 3:**
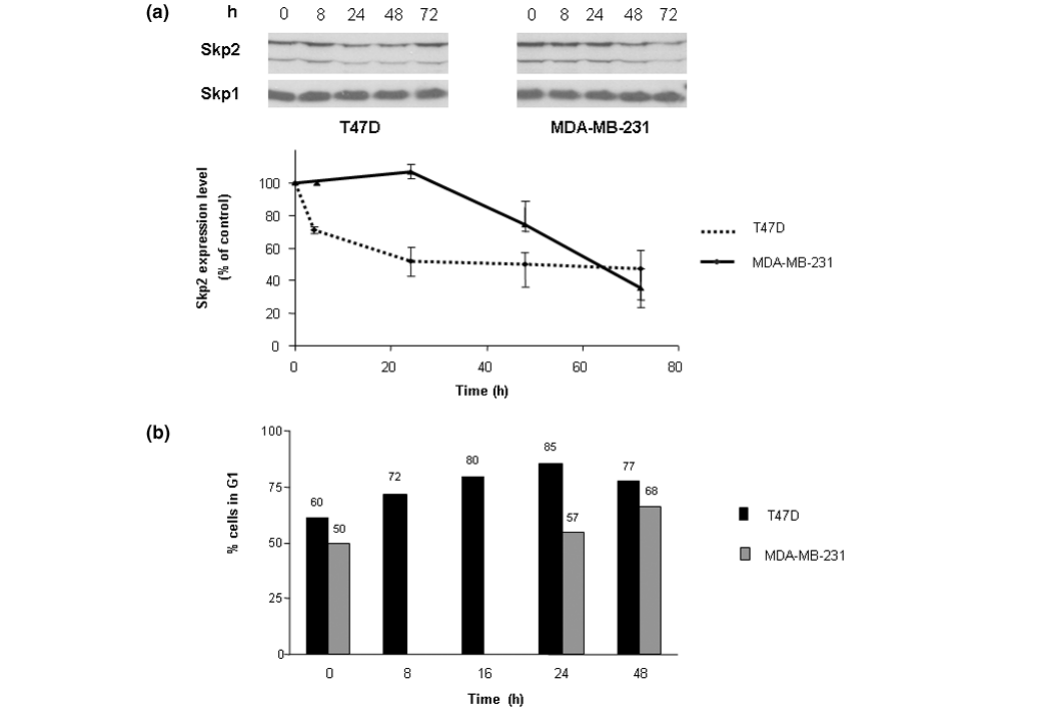
The effect of rapamycin on Skp2 protein levels and cell cycle distribution. (**a**) The effect of rapamycin on Skp2 protein levels. The breast cancer cell lines T47D and MDA-MB-231 were treated with rapamycin (20 nM) for different time periods and the expression of the protein was determined by western blot analysis. Skp1 was used as a loading control. (**b**) The time-dependent effect of rapamycin on G1 distribution in T47D and MDA-MB-231 cells. Cells were treated with rapamycin (20 nM) for different time periods and the percentage of cells in G1 was determined by FACS analysis using Modfit LT 2.0 software.

Recent studies have shown that activation of the PI3K/Akt pathway and its downstream mTOR signaling pathway promote, at least in part, the proliferation rate of breast cancer by down-regulating p27 nuclear protein levels. Rapamycin, in turn, was shown to inhibit this effect and stabilize p27 levels, but whether this effect results from decreased ubiquitin-mediated degradation is unknown. To examine the effect of rapamycin on the expression of Skp2, we initially tested this effect in T47D, a breast cancer cell line that showed high sensitivity to rapamycin in our initial experiments. Cells were treated with rapamycin at a concentration of 20 nM for different time periods up to 72 h and subjected to western blot analysis. Treatment with rapamycin significantly decreased Skp2 at 24 h (Figure 3a), a time point that preceded the initiation of cell proliferation arrest (Figure 1b). To examine whether this association was valid in other cell lines, we examined the effect of rapamycin on cell proliferation and Skp2 expression in MDA-MB-231, a breast cancer cell line that has shown delayed sensitivity to rapamycin. Because Skp2 levels change during the cell cycle (being normally highest in the S-phase and lowest in G1) we cultured the cells in different media conditions until similar growth rates were reached for the two cell lines. The effect of rapamycin on Skp2 expression in MDA-MB-231 cells was evident only after 48 h (Figure 3a), but again, it preceded the initiation of cell growth inhibition in this cell line (Figure 1b).

Agents that cause a block in G1 can affect cell cycle progression up to a full cell cycle before any difference in cell number is apparent, since unaffected cells in S and G2/M phases can complete a cell cycle and divide before being arrested in G1 in the subsequent cell cycle. We therefore examined the cell cycle distribution over the first 24 h for T47D cells and at 24 and 48 h for MDA-MB-231 cells (Figure 3b). At 8 h 72% of T47D cells were arrested in G1, increasing to 80% and 85% at 16 h and 24 h, respectively. At 24 h only 57% of MDA-MB-231 cells were arrested in G1 (compared to 50% in untreated cells), but the percentage of cells arrested in G1 increased to 68% at 48 h. Taken together, these results suggest that the negative effect of rapamycin on Skp2 expression has an important role in rapamycin-mediated cell growth arrest.

**Figure 4:**
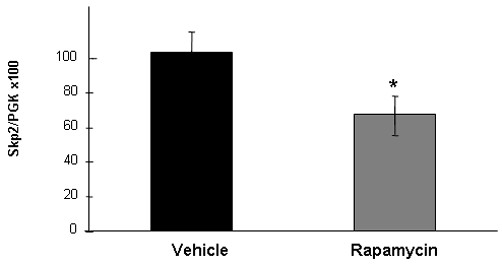
The effect of rapamycin on Skp2 mRNA levels. T47D cells were treated with rapamycin (20 nM) for 8 h and the change in Skp2 mRNA levels was determined by real-time RT-PCR. PGK, phosphoglycerate kinase.

**Figure 5:**
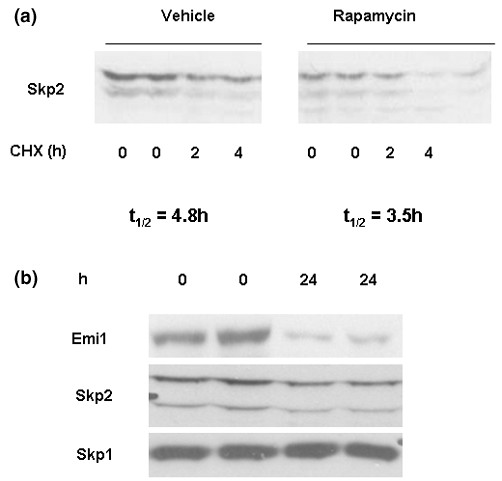
The effect of rapamycin on Skp2 degradation. (**a**) The effect of rapamycin on Skp2 degradation rate. T47D cells were treated with rapamycin (20 nM) or DMSO (0.02%) for 24 h followed by treatment with cycloheximide (CHX; 100 μg/ml) for the indicated time periods. Levels of Skp2 protein levels were determined by western blot analysis. (**b**) The effect of rapamycin on Emi1 protein levels. T47D cells were treated with rapamycin (20 nM) and levels of Emi1 were determined by western blot analysis. Skp1 was used as a loading control.

**Figure 6:**
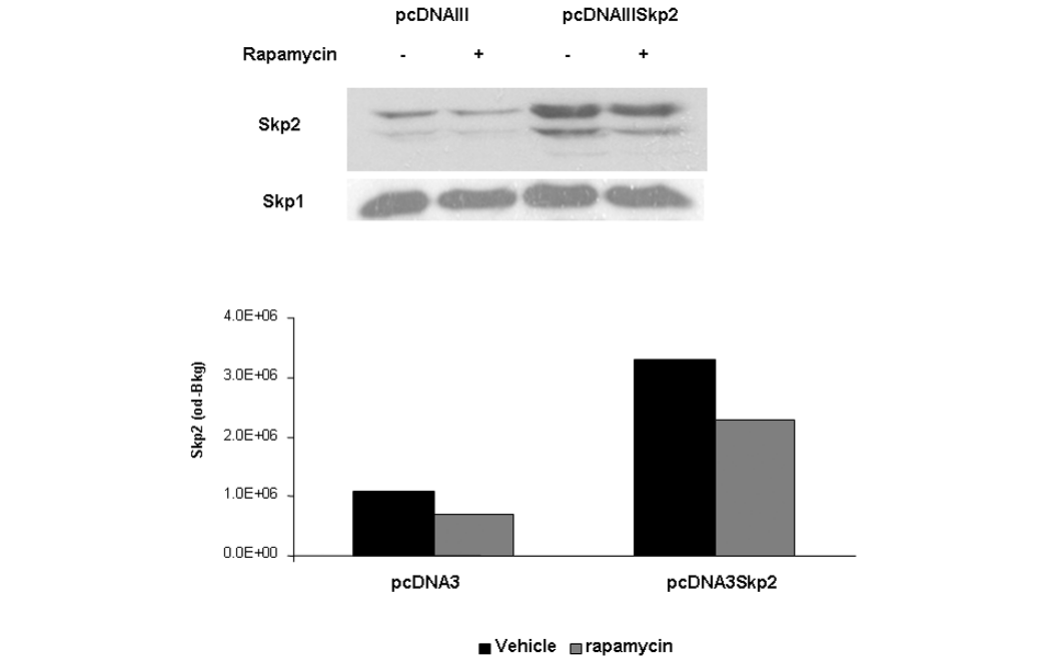
T47D cells were transiently transfected with a Skp2 insert or an empty plasmid (vehicle) and treated with rapamycin (20 nM) for 48 h. Levels of Skp2 protein levels were determined by western blot analysis. Optic Density (od) of the Skp2 band as measured by chemiluminesence – blot background (Bkg).

Recent evidence suggests that Skp2 is encoded by an oncogene that may be overexpressed in a large variety of cancers, including breast cancer. More recently, it was found that Skp2 levels may also be regulated at the post-transcriptional level by its rate of ubiquitin-mediated degradation, regulated by its specific ubiquitin ligase APC/C [12]. Thus, it was important to explore the mechanisms by which rapamycin down-regulates Skp2 expression in breast cancer. In order to examine whether the decrease in Skp2 protein levels is due to inhibition of transcriptional activation, we subjected T47D cells to 20 nM rapamycin for 8 h and measured mRNA levels using real-time RT-PCR. A significant decrease in Skp2 mRNA levels (28%) was measured in rapamycin-treated cells compared to control cells (Figure 4). No further decrease in Skp2 mRNA levels was observed at later time points (data not shown). To examine whether rapamycin affected the degradation rate of Skp2, we next exposed cells to the protein synthesis inhibitor cycloheximide and measured the decay in Skp2 protein levels. The half-life of Skp2 in vehicle-treated cells was 4.6 h whereas in rapamycin-treated cells it was 3.5 h (a decrease of 24%) (Figure 5a). Previous studies showed that accelerated degradation of Skp2 may result from the alterations in the expression of Emi1, an inhibitory protein that binds to APC/C and renders it inactive [12]. As shown in Figure 5b, Emi1 levels were down-regulated in rapamycin-treated T47D cells compared to controls. Taken together, these results suggest that rapamycin leads to an accelerated rate of Skp2 degradation, which may be associated with increased activation of APC\C. To further examine whether rapamycin affects Skp2 regulation at the translational level, we transiently transfected cells with a plasmid containing a Skp2 insert; 24 h after the transfections, cells were treated with rapamycin or a vehicle for 48 h. Skp2 protein levels were significantly higher in Skp2-transfected cells compared to cells transfected with an empty plasmid (Figure 6). When treated with rapamycin, the reduction in Skp2 protein levels was similar in both cells (about 30%), suggesting that rapamycin may also regulate Skp2 protein levels at the post-transcriptional level.

## Discussion

Recent studies have provided evidence that alterations in the expression of different cell cycle regulatory proteins may have a significant impact on the progression and outcome of cancer in general and in breast cancer in particular. Among these cell cycle regulatory proteins, the oncogenic role of Skp2 in breast cancer has been clearly demonstrated [11,29]. Through mechanisms that are yet not completely understood, Skp2 is overexpressed in some cancers and is associated with poor disease free and overall survival. Skp2 is the ubiquitin ligase subunit that targets p27 for degradation and is the major determinant of p27 deregulation in cancer. Because of its important role as an inhibitor of Cdks at G1, down-regulation of p27 tumor levels allows uncontrolled tumor proliferation. Recently, other roles for Skp2 were discovered that may effect cell cycle progression. For example, it was discovered that Skp2 regulates the rate of degradation of the Cdk inhibitor p21 and of the forkhead transcription factor FOXO-1, two other cell cycle regulatory proteins that play important roles in cancer progression [30,31]. Thus, the identification of novel therapeutic interventions that may down-regulate the expression of Skp2 in cancer may potentially lead to a significant decrease in cancer progression and control of the disease. Unfortunately, specific drugs that target Skp2 are unavailable at present and it is, therefore, important to identify commonly used drugs that have inhibitory effects on Skp2 expression.

The results of the present study show that specific inhibition of the mTOR pathway by rapamycin may significantly down-regulate Skp2 levels in rapamycin-sensitive breast cancer cells. This effect may explain in part the findings of stabilization of p27 levels and cell-cycle arrest at G1 by rapamycin. These results are important for several reasons. First, these findings provide additional insight into the mechanisms of action by which rapamycin arrests cell growth in breast cancer. Previous studies have shown that activation of S6K1 and 4E-BP1 enhances the translation of critical mRNAs that are involved in cell cycle progression and cell proliferation, while inactivation and dephosphorylation of these proteins inhibits this process, leading to cell cycle arrest in G1 [24,25]. The increase in p27 levels by rapamycin observed in a number of studies could theoretically be secondary to cell cycle arrest at G1. However, our results show that this effect may result, at least in part, from direct down-regulation of Skp2 by rapamycin. The decrease in Skp2 levels preceded that of cell growth inhibition and G1 arrest in both cell lines, suggesting that this specific effect of rapamycin contributes significantly to cell growth arrest. Second, constitutive activation of the PI3K/Akt pathway frequently occurs in breast cancer and some of its oncogenic effects are mediated through the mTOR pathway. This is especially true in PTEN-deficient tumors or tumors overexpressing Her-2/neu receptors, which were found to activate this pathway and were also commonly associated with Skp2 overexpression in different cancers [9]. Thus, it seems that rapamycin treatment in these tumors should be most beneficial. However, not all breast cancer cells *in vitro *and tumors *in vivo *respond equally to rapamycin and clinically determining the sensitivity to this drug is of great difficulty. For example, the PI3K/Akt/mTOR pathway is regulated by PTEN, but not all PTEN-deficient cells are rapamycin sensitive. Moreover, in our study we did not find a relationship between the levels of Skp2 expression and sensitivity to rapamycin. Thus, the issue of which subsets of tumors overexpressing Skp2 may respond the most to rapamycin is at present unclear. Finally, we show here for the first time the possible involvement of the APC/C in the regulation of Skp2 abundance in breast cancer cells. We found that treatment with rapamycin enhanced Skp2 protein degradation and that this was associated with down-regulation of Emi1, the inhibitor of the APC/C. Thus, these results suggest that Skp2 deregulation in breast cancer may also be attributed to stabilization of the protein through decreased degradation rate, and not only from increased transcription.

## Conclusion

The results of the present study provide additional insights into the mechanisms of action of rapamycin on cell cycle arrest in breast cancer cells through direct down-regulation of Skp2 expression. Rapamycin inhibited the transcription of Skp2 and at the same time led to protein destabilization and enhanced degradation rate. Because Skp2 plays an important role in tumor progression in breast cancer and clinical outcome, these results suggest that rapamycin may be of benefit in cancers expressing high Skp2 levels.

## Abbreviations

4E-BP1 = 4E-binding protein 1; APC/C = anaphase promoting complex/Cdh1; Bkg = blot background; Cdk = cyclin dependent kinase; DMSO = dimethyl sulfoxide; Emi1 = early mitotic inhibitor 1; FACS = fluorescence-activated cell sorting; mTOR = mammalian target of rapamycin; od = optic density ; PBS = phosphate-buffered saline; PGK = phosphoglycerate kinase; PI3K = phosphoinositol 3' kinase; PMSF = phenylmethanesulfonyl fluoride; PTEN = phosphatase and tensin homologue deleted from chromosome 10; RT-PCR = reverse-transcription PCR; Skp = S phase kinase protein; TBST = Tris Buffer Saline with 0.1% Tween 20.

## Competing interests

The authors declare that they have no competing interests.

## Authors' contributions

MS carried out the immunoblot analysis, FACS analysis, transient transfections of constructs, performed the statistical analysis and participated in drafting the manuscript. EK carried out the proliferation studies and part of the immunoblot analysis. TR performed the mRNA real-time RT-PCR studies and degradation assays. DDH conceived of the study, participated in the design of the study, participated in the sequence alliance and helped draft the manuscript. All authors read and approved the final version.

## References

[B1] Tsihlias J, Kapusta L, Slingerland J (1999). The prognostic significance of altered cyclin-dependent kinase inhibitors in human cancers. Annu Rev Med.

[B2] Hengst L, Reed SI (1996). Translational control of p27^Kip1 ^accumulation during the cell cycle. Science.

[B3] Pagano M, Tam SW, Theodoras AM, Beer-Romano P, Del Sal G, Chau V, Yew PR, Draetta GF, Rolfe M (1995). Role of the ubiquitin-proteasome pathway in regulating abundance of the cyclin-dependent kinase inhibitor p27. Science.

[B4] Carrano AC, Eytan E, Hershko A, Pagano M (1999). Skp2 is required for ubiquitin-mediated degradation of the Cdk inhibitor p27. Nat Cell Biol.

[B5] Sutterluty H, Chatelain E, Marti A, Wirbeluauer C, Senften M, Muller U, Krek W (1999). p45Skp2 promotes p27Kip1 degradation and induces S phase in quiescent cells. Nat Cell Biol.

[B6] Deshaies RS (1999). SCF and Cullin/Ring H2 based ubiquitin ligases. Annu Rev Cell Dev Biol.

[B7] Zhang H, Kobayashi R, Galaktionov K, Beach D (1995). p19^Skp1 ^and p45^Skp2 ^are essential elements of the cyclin A-Cdk2 S phase kinase. Cell.

[B8] Hershko D, Bornstein G, Ben-Izhak O, Carrano A, Pagano M, Krausz MM, Hershko A (2001). Inverse relation between levels of p27(Kip1) and of its ubiquitin ligase subunit Skp2 in colorectal carcinomas. Cancer.

[B9] Yang G, Ayala G, Marzo AD, Tian W, Frolov A, Wheeler TM, Thompson TC, Harper JW (2002). Elevated Skp2 protein expression in human prostate cancer: association with loss of the cyclin-dependent kinase inhibitor p27 and PTEN and with reduced recurrence-free survival. Clin Cancer Res.

[B10] Gstaiger M, Jordan R, Lim M, Catzavelos C, Mestan J, Slingerland J, Krek W (2001). Skp2 is oncogenic and overexpressed in human cancers. Proc Natl Acad Sci USA.

[B11] Signoretti S, Di Marcotullio L, Richardson A, Ramaswamy S, Issac B, Rue M, Monti F, Loda M, Pagano M (2002). Oncogenic role of the ubiquitin ligase subunit Skp2 in human breast cancer. J Clin Invest.

[B12] Bashir T, Dorrello NV, Amador V, Guardavaccaro D, Pagano M (2004). Control of the SCF (Skp2-Cks1) ubiquitin ligase by the APC/C(Cdh1) ubiquitin ligase. Nature.

[B13] Wei W, Ayad NG, Wan Y, Zhang GJ, Kirschner MW, Kaelin WG (2004). Degradation of the SCF component Skp2 in cell-cycle phase G1 by the anaphase-promoting complex. Nature.

[B14] Liang J, Zubovitz J, Petrocelli T, Kotchetkov R, Connor MK, Han K, Lee JH, Ciarallo S, Catzavelos C, Beniston R (2002). PKB/Akt phosphorylates p27, impairs nuclear import of p27 and opposes p27-mediated G1 arrest. Nat Med.

[B15] Shin I, Yakes FM, Rojo F, Shin NY, Balin AV, Baselga J, Arteaga CL (2002). PKB/Akt medoates cell cycle progression by phosphorylation of p27 at theronine 157 and modulation of its cellular localization. Nat Med.

[B16] Bos JL (1989). ras oncogenes in human cancers: A review. Cancer Res.

[B17] Di Cristofano A, Pandolfi PP (2000). The multiple roles of PTEN in tumor suppression. Cell.

[B18] Tsai EM, Wang SC, Lee JN, Hung MC (2001). Akt activation by estrogen in estrogen negative breast cancer cells. Cancer Res.

[B19] Yang HY, Shao R, Hung MC, Lee MH (2001). P27Kip1 inhibits HER2/neu-mediated cell growth and tumorigenesis. Oncogene.

[B20] Lenferink AE, Busse D, Flanagan WM, Yakes FM, Arteaga CL (2001). Erb2/neu kinase modulates cellular p27 and cyclin D1 through multiple signaling pathways. Cancer Res.

[B21] Murrillo H, Huang H, Schmidt LJ, Smith DI, Tindall DJ (2001). Role of PI3K signaling in survival and progression of LNCaP prostate cancer cells to androgen refractory state. Endocrinology.

[B22] Gesbert F, Sellers WR, Signoretti S, Loda M, Griffin JD (2000). BCR/ABL regulates expression of the cyclin dependent kinase inhibitor p27Kip1 through the phosphatidylinositol 3-Kinase/AKT pathway. J Biol Chem.

[B23] Pene F, Claessens YE, Muller O, Vigiue F, Mayeux P, Dreyfus F, Lacombe C, Bouscary D (2002). Role of the phosphatidylinositol 3-kinase/Akt and mTOR/P70S6-kinase pathways in the proliferation and apoptosis in multiple myeloma. Oncogene.

[B24] Hidalgo M, Rowinsky EK (2000). The rapamycin-sensitive signal transduction pathway as a target for cancer therapy. Oncogene.

[B25] Mills GB, Lu Y, Kohn EC (2001). Linking molecular therapeutics to molecular diagnostics: inhibition of the FRAP/RAFT/TOR components of the PI3K pathway preferentially blocks PTEN mutant cells in vitro and in vivo. Proc Natl Acad Sci USA.

[B26] Raymond E, Alexandre J, Faivre S, Vera K, Materman E, Boni J, Leister C, Korth-Bradley J, Hanauske A, Armand JP (2004). Safety and pharmacokinetics of escalated doses of weekly intravenous infusion of CCI-779, a novel mTOR inhibitor, in patients with cancer. J Clin Oncol.

[B27] Meric-Bernstam F, Mills GB (2004). Mammalian target of rapamycin. Semin Oncol.

[B28] Noh WC, Mondesire WH, Peng J, Jian W, Zhang H, Dong J, Mills GB, Meric-Bernstam F (2004). Determinants of rapamycin sensitivity in breast cancer cells. Clin Cancer Res.

[B29] Slotky M, Shapira M, Ben-Izhak O, Linn S, Futerman B, Tsalic M, Hershko DD (2005). The expression of the ubiquitin ligase subunit Cks1 in human breast cancer. Breast Cancer Res.

[B30] Borenstein G, Bloom J, Sitry-Shevah D, Nakayama K, Pagano M, Hershko A (2003). Role of the SCFSkp2 ubiquitin ligase in the degradation of p21Cip1 in S phase. J Biol Chem.

[B31] Huang H, Regan KM, Wang F, Wang D, Smith DI, van Deursen JM, Tindall DJ (2005). Skp2 inhibits FOXO1 in tumor suppression through ubiquitin-mediated degradation. Proc Natl Acad Sci USA.

