# Correction to: The mTOR inhibitor rapamycin down-regulates the expression of the ubiquitin ligase subunit Skp2 in breast cancer cells

**DOI:** 10.1186/s13058-018-1000-4

**Published:** 2018-07-09

**Authors:** Ma’anit Shapira, Eli Kakiashvili, Tzur Rosenberg, Dan D. Hershko

**Affiliations:** 1 Department of Surgery A, Rambam Medical Center, 31096 Haifa, Israel; 2 Technion – Israel Institute of Technology, 31096 Haifa, Israel; 3 Department of Surgery B, Rambam Medical Center, 31096 Haifa, Israel; 4 Breast Health Institute, Rambam Medical Center, 31096 Haifa, Israel

## Correction

After the publication of this work [1], an error was noticed in Fig. 2b, Fig. 3a and Fig. 5b. The Skp1 loading control was accidentally duplicated. We apologize for this error, which did not affect any of the interpretations or conclusions of the article.
